# Generative AI and student learning performance in medical higher education: a social cognitive theory perspective

**DOI:** 10.3389/fmed.2026.1807322

**Published:** 2026-05-29

**Authors:** Muhammad Azeem Ashraf, Muhammad Aftab, Muhammad Mohsin, Sohail Maqbool, Muhammad Hanif

**Affiliations:** 1 Institute of Educational Sciences, Hunan University, Changsha, China; 2 Faculty of Management Sciences, University of Okara, Okara, Pakistan; 3 Department of Business and Management, University of Sultan Zain ul Abidin, Kuala Terengganu, Malaysia; 4 Faculty of Management Sciences, National University of Modern Languages, Islamabad, Pakistan

**Keywords:** cognitive engagement, fairness and ethics, generative AI, learning performance, perceived learning outcomes, technology self-efficacy

## Abstract

**Purpose:**

This study examines the impact of Generative Artificial Intelligence (GAI) on medical students' learning performance in higher education. Drawing on Social Cognitive Theory, it investigates the mediating roles of technology self-efficacy, perceived learning outcomes, and cognitive engagement, as well as the moderating role of fairness and ethical perceptions in AI-supported learning environments.

**Methods:**

A cross-sectional quantitative design was employed, with data collected from 326 medical higher education students in Pakistan using validated survey instruments. Structural equation modeling (SEM) with SmartPLS 4.0 was applied to test direct, mediating, and moderating relationships among the study variables.

**Results:**

The findings reveal that GAI-based technologies (GAT) exert a significant positive direct effect on students' learning performance and indirect effects through technology self-efficacy, perceived learning outcomes, and cognitive engagement. Technology self-efficacy and cognitive engagement emerged as strong mediators, while perceived learning outcomes further supported academic motivation and achievement. Moderation analysis demonstrated that perceptions of fairness and ethics significantly strengthen the positive effects of these mediators on learning performance, underscoring the importance of ethically grounded AI implementation.

**Conclusion:**

The study provides empirical evidence that GAI can enhance learning performance in medical higher education when supported by strong self-efficacy, engagement, and positive learning perceptions. Importantly, ethical and fair AI practices amplify these benefits. The findings offer practical implications for educators and policymakers seeking to integrate generative AI responsibly and effectively into medical education.

## 1 Introduction

Medical colleges and universities are at the forefront of developing future leaders, innovators, and professionals capable of tackling complex global challenges (1). Such institutions also offer students the chance to acquire both academic, professional, and personal competencies that are important in achieving success. Nevertheless, some limitations that impede the progress of students include complicated course designs, poor academic support, and the lack of access to educational materials (2). Thus, academic support has gradually turned into a strategic initiative of medical colleges to not only positively affect the learning outcomes of students but also to increase retention rates and persistence rates of the institution (3). These are done with the aim of turning universities into those environments where students are engaged, healthy, and successful. The previous studies point out the growing need among students for personalized academic and emotional support (1, 4). Johnson et al. (5) discovered that 90 percent of students have academic challenges, including stress and anxiety, and a lack of knowledge on how to plan in the future, but few students indicate they get adequate support. In addition, gender variation influences help-seeking, and women tend to request and receive academic assistance more often than men (6).

Generative Artificial Intelligence (GAI) has emerged as a crucial element capable of addressing various challenges by transforming the learning process. Systems based on GAI, such as AI chatbots and intelligent tutoring systems, function as adaptive teaching assistants that deliver personalized instruction, feedback, and support (7, 8). Unlike traditional learning technologies, GAI facilitates the creation of personalized and dynamic learning environments that encourage self-directed and active learning (9). These systems assist students in planning their studies, setting goals, and conducting evaluations through generative modeling techniques (10, 11). As development progresses, GAI systems are increasingly utilized to monitor learning progress and enhance instructional quality through integrated multimodal data, analytics, and feedback mechanisms (12, 13). The contribution of GAI to the learning outcomes of Medical students hinges on its ability to enhance cognitive processing and attention. Grounded in cognitive theory, GAI can deliver complex content in structured, multimodal formats that reduce extraneous cognitive load, thereby improving retention and comprehension (7). For instance, GAI's context-based use of examples and interactive feedback in science education reduces mental load and fosters deeper learning (14). Likewise, multimedia applications with language understanding capabilities, such as AI-generated poetry videos, support language acquisition by integrating text and visual elements to facilitate cognitive processing (15).

Technology self-efficacy (TSE), defined as students' confidence in their ability to effectively utilize AI technologies, is a critical factor influencing the efficacy of GAI-based learning (16, 17). TSE impacts students' engagement with technology, how they handle challenges, and their motivation to learn, which ultimately affects perceived learning outcomes (PLO) and cognitive engagement (CE). When students believe they can operate AI systems, they are more likely to engage in the cognitive process and achieve better learning results (18). These mediating variables align with the cognitive and motivational processes emphasized in social cognitive theory, which posits that learning is shaped by the reciprocal interaction of personal, behavioral, and environmental factors (19).

Nonetheless, fairness and ethics (FE) are essential to the effectiveness of GAI-based technologies (GAT). Applying AI in ethical contexts promotes clarity, responsibility, and fairness in the learning process (20). Fairness in AI involves treating students equitably regardless of their background or abilities, while ethical design ensures responsible data use and learner protection (21, 22). It is important to incorporate fairness and ethics as moderating factors in the model, as these concepts enhance trust, acceptance, and promote equitable learning outcomes.

Technology adoption in educational contexts has often been explained using models such as the Technology Acceptance Model (TAM) and the Unified Theory of Acceptance and Use of Technology (UTAUT). TAM posits that users' acceptance of technology is primarily determined by perceived usefulness and perceived ease of use, which influence behavioral intention and actual usage (23). UTAUT extends this perspective by incorporating additional determinants such as performance expectancy, effort expectancy, social influence, and facilitating conditions (24). While these models are effective in explaining technology adoption behavior, they provide limited insight into the cognitive and motivational processes that influence learning performance.

Although the literature on artificial intelligence in education is rapidly expanding, there is limited empirical research specifically examining GAT and the processes through which they influence student learning outcomes in higher education. Most existing studies focus on traditional AI systems and are grounded in technology adoption models such as TAM and UTAUT which offer limited insight into the cognitive and motivational mechanisms underlying learning performance. Furthermore, the mediating roles of technology self-efficacy, perceived learning outcomes, and cognitive engagement are understudied, particularly within medical higher education, where learning involves significant cognitive and ethical challenges. Additionally, issues of fairness and ethics, key boundary conditions impacting the effectiveness of generative AI systems, remain underexplored. This study aims to address these gaps by adopting a framework based on social cognitive theory. The objective is to investigate how GAT influence the learning performance of medical higher education students, while also examining the mediating effects of technology self-efficacy, perceived learning outcomes, and cognitive engagement, as well as the moderating roles of fairness and ethics within the Social cognitive theory framework.

## 2 Literature review

### 2.1 Social cognitive theory

Social Cognitive Theory (SCT) represents an extension of social learning theory, offering a cognitive perspective that elucidates human behavior as an intricate interplay among personal, environmental, and behavioral factors (19). This integrative framework synthesizes elements of cognitive, behavioral, and emotional theories of behavior change, emphasizing the significance of observational learning, reinforcement, self-regulation, and self-efficacy. It is among the most extensively applied theories for understanding health-related behaviors (25). According to SCT, there exists a reciprocal relationship among the individual, their environment, and behavior, with these elements being dynamically interconnected and mutually influential. Such interrelations underpin behavior development and serve as targets for intervention strategies (19). Bandura's formulation of SCT underscores the importance of environmental influences, individual characteristics, and behavioral patterns in learning processes and technology adoption. Perceived institutional support constitutes an environmental factor that influences students' perceptions and behaviors toward AI-supported learning by fostering an enabling environment that facilitates technology use. This support, including training provisions and resource availability, enhances students' self-efficacy regarding AI tools, subsequently improving their perception of the technology's utility. In the context of this study, SCT is applied to examine the interaction among the individual factor of technology self-efficacy, the environmental factor of perceived institutional support, and behavioral outcomes such as students' perceptions of AI-supported learning.

### 2.2 Learning performance and GAT

Adaptive artificial intelligence, driven by user interaction, can support college learners effectively. The chatbot-based learning system aims to achieve motivational and instructional objectives by offering a conversational interface, clear learning outcomes, and enabling students to learn flexibly on their own schedule (26). Maheshwari (27) highlights that ChatGPT differs from other chatbots due to its rapid text-generation capabilities. According to the Social Cognitive Theory (SCT), generative AI can enhance educational outcomes by fostering an environment conducive to information exchange, interactive training, and immediate feedback. Such an environment promotes greater student engagement, motivation, and interpersonal connection, leading to improved learning outcomes and increased productivity (28). Evidence indicates that AI can effectively stimulate learner motivation to acquire knowledge (17, 29). Research has shown that ChatGPT and similar generative AI chatbots can increase students' interest in learning (26, 30). The use of these AI tools in the learning process has been associated with heightened motivation among college students (31). The advanced language processing capabilities of generative AI and ChatGPT facilitate more efficient learning by engaging students and offering unique, personalized experiences. Students are particularly attracted to ChatGPT's interactive features, which encourage active participation, discussion, and inquiry (30). Consequently, it is hypothesized that:

***H1****. GAT have a significant influence on the learning performance of medical higher education students*.

### 2.3 The mediating role of cognitive engagement

Constructivism conceptualizes cognitive engagement in learning as the degree to which students exert mental effort in their activities, such as applying their knowledge and employing cognitive strategies to complete tasks (18). Conversely, cognitive involvement pertains more to mental processes like thinking, planning, problem-solving, and decision-making (32, 33). Cognitive engagement is frequently discussed within educational settings to assess students' active participation, including their thinking, problem-solving, and engagement with substantial amounts of information. Enhancing learning performance is often associated with higher levels of cognitive involvement in the learning process (34). According to the Interactive Theory, the degree of student interaction with GAT influences their level of mental engagement, which in turn impacts learning outcomes. This theory emphasizes that the stimuli students encounter during interaction are critically important (35). Students are more likely to engage when they have access to GAT enabling real-time interaction and immediate feedback.

However, it is important to recognize that effective interaction with GAT requires a certain level of prior knowledge and critical thinking ability. While generative AI systems often rely on users' ability to iteratively refine prompts to obtain optimal outputs, this assumption may not hold for novice learners. Students in the early stages of their education may lack the domain knowledge necessary to critically evaluate AI-generated responses, identify inaccuracies, or formulate effective prompts. As a result, they may accept AI outputs uncritically or fail to engage in deeper cognitive processing. Therefore, the effectiveness of GAT in fostering cognitive engagement is contingent upon learners' prior knowledge, digital literacy, and metacognitive skills. Instructional scaffolding, including explicit guidance on prompt formulation, critical evaluation of AI outputs, and iterative learning strategies, becomes essential in supporting students' effective use of AI tools. Educators play a crucial role in facilitating this process by helping learners develop the skills necessary to engage meaningfully with AI-supported learning environments.

Additionally, GAI can supply numerous images, facts, and examples that stimulate deeper thinking and exploration. For instance, detailed responses from GAT may capture students' interest and curiosity, thereby increasing their engagement in the learning process. It is important to emphasize that the quality and nature of inputs provided to GAT significantly influence the content it produces (36). For instance, when querying GAT on a particular subject, it may deliver an excellent response; however, if insufficient details are provided, the responses tend to be brief and biased (22). To ensure accurate answers, students should repeatedly refine and rephrase their questions. This iterative process can effectively engage young learners. Additionally, GAT may generate false or inaccurate references and exhibit confident but flawed responses (37). It is essential for students not to accept information at face value but to critically evaluate the materials they encounter. Continuous interaction with these materials fosters deeper, more reflective learning. Consequently, this paper hypothesizes:

***H*2**. *Cognitive engagement mediates the relationship between GAT and learning performance*

### 2.4 Mediating role of perceived learning outcome

Students report increased happiness when using GAT, which they believe enhance their learning by boosting their sense of value and ease of use (38). Both GAI and mobile learning positively impact perceived learning outcomes, with self-competence serving as a key mediator between these factors (39, 40). Personal learning environments influence individuals' ability to engage positively with AI-enabled e-learning, thereby affecting perceptions of its ease and value (41). Intrinsic motivation plays a significant role in the relationship between perceived AI learning and computational thinking, underscoring the importance of student interest (40). Additionally, intrinsic motivation may further influence AI's impact on student performance. This study demonstrates the complex interplay between external support and internal drive. Moreover, intelligent learning systems facilitate the integration of AI into instruction, contributing to improved academic outcomes (42). Consequently, it is hypothesized that:

***H*3**. *Perceived learning outcome mediates the relationship between GAT and learning performance*.

### 2.5 The mediating role of self-efficacy

Self-efficacy theory describes an individual's confidence in their ability to perform specific tasks, which involves assessing their capacity to complete such tasks successfully. This self-efficacy develops gradually through experiences, observations, and interactions. In educational contexts, students with higher self-efficacy tend to achieve better academic outcomes, as they believe in their ability to attain their goals (43). This paper proposes that GAT can serve as effective aids for students, potentially enhancing their self-efficacy. Notably, GAI demonstrates remarkable proficiency in handling complex tasks, including article composition (44), storytelling, poetry, essay writing (45), image generation (46), and generating or debugging computer code (47).

GAT may generate extraordinary levels of executive functioning and creativity in interactions that are often beyond human capabilities (48). When working with such technologies, students can recognize their potential to produce innovative and satisfying content or manage complex tasks using GAT. Their ability to achieve objectives with GAI's assistance can serve as a measure of their competence (18). In this context, interaction with GAI may enhance students' self-efficacy. Furthermore, GAI has the capacity to tailor content according to students' individual understanding levels and academic backgrounds, providing a personalized learning experience. Since students' knowledge bases vary across subjects (18), they may require multiple explanations and examples to grasp abstract or novel concepts, which can be labor-intensive for instructors (49). Educators must monitor emerging trends and students' cognitive load to deliver effective instruction aligned with learners' proficiency levels (22). However, time and resource constraints may limit teachers' ability to meet all student needs, a challenge that GAI can help address (49). Interacting with GAI can foster a perception of personalized learning experiences among students (49), enabling them to understand complex information more efficiently (50). This interaction may boost students' confidence in their learning abilities, thereby increasing their self-efficacy. Consequently, it is hypothesized that:

***H*4**. Self-efficacy mediates the relationship between GAT *and learning performance*.

### 2.6 Moderating role of fairness and ethics

The study by Charlwood and Guenole (51) indicates that fairness in e-learning necessitates equal and equitable treatment of all students, with evaluations and assessments conducted in an unbiased manner. Ashok et al. (52) highlight that online learning ethics involve adhering to moral principles, maintaining confidentiality, and demonstrating sincerity and trustworthiness in online interactions. Upholding these fundamental principles fosters a supportive and effective e-learning environment that promotes both learning and collaboration (53). The ethics and justice embedded in SCT, facilitated by AI technologies, enhance learning outcomes through observational methods. Fairness and ethical conduct significantly influence learner performance and motivation, thereby creating a just learning environment that boosts productivity (29). The integration of chatbots and large language models such as ChatGPT contributes to a fair and ethical e-learning process, supporting students' ability to learn more effectively at higher education levels. Nonetheless, some researchers suggest that ChatGPT may pose ethical challenges to academic integrity due to its misuse (54, 55). Conversely, most studies indicate that LLMs and chatbots promote ethical considerations and stimulate student creativity by providing personalized support and assistance in editing and refining their work (22).

***H*5**. *Fairness & ethics moderate the association between cognitive engagement and learning performance*.
***H*6**. *Fairness & ethics moderate the relationship between perceived learning outcome and learning performance*.
***H7*. ***Fairness & ethics moderate the connection between self-efficacy and learning performance*.

#### 2.6.1 Research framework

**Figure 1:**
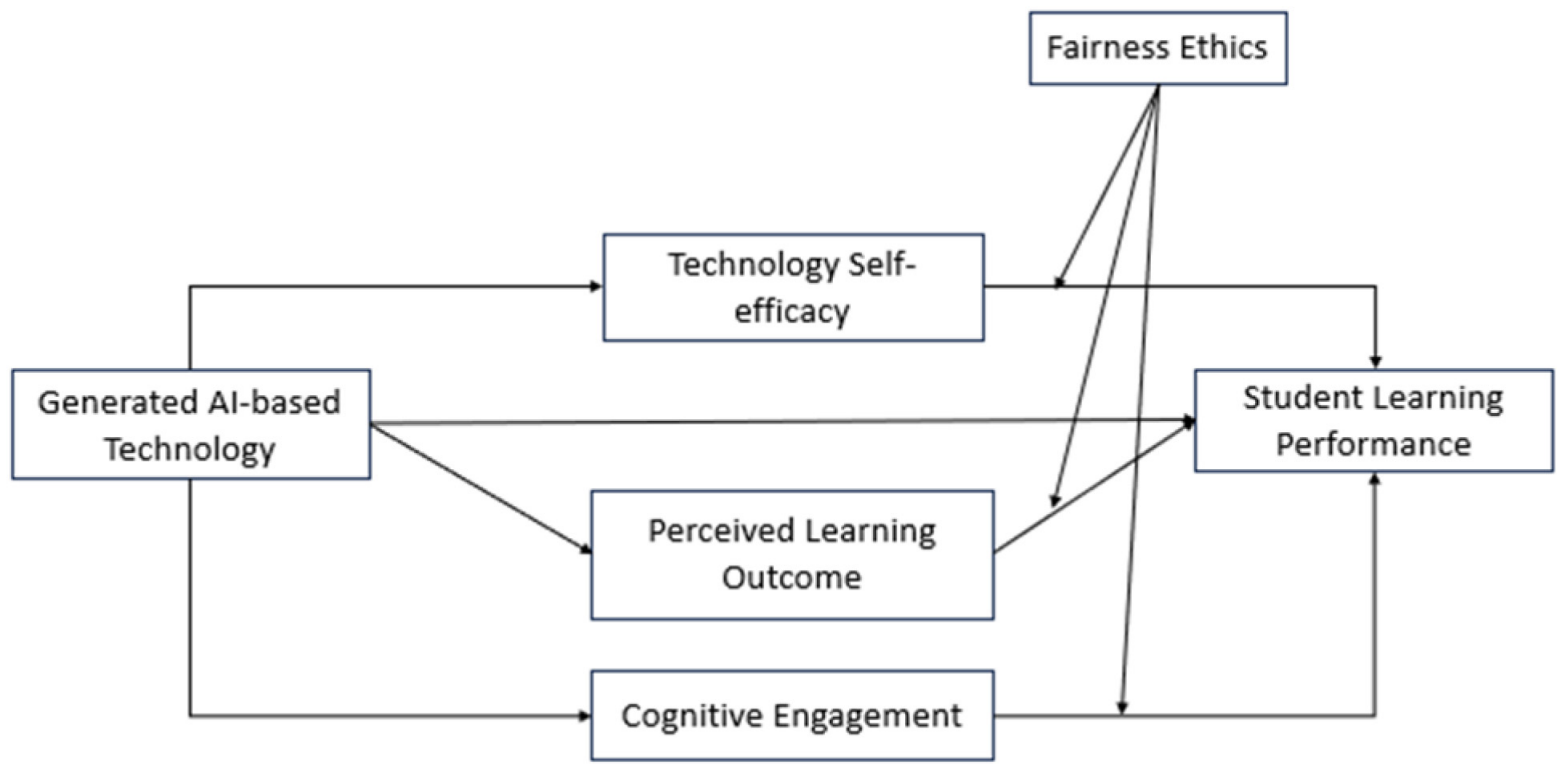
Research Framework.

Figure 1 presents the conceptual framework of this study, grounded in Social Cognitive Theory. The model illustrates the direct effect of generative AI technologies on student learning performance, as well as indirect effects mediated by cognitive engagement, perceived learning outcomes, and technology self-efficacy. Additionally, fairness and ethics are modeled as moderating variables influencing the strength of these relationships.

## 3 Research methodology

### 3.1 Research design

This study employed a cross-sectional quantitative research design to systematically explore medical higher education students' perceptions of AI-enhanced learning. Specifically, the study investigated the relationships among generative AI technologies, technology self-efficacy, cognitive engagement, fairness and ethics perceptions, perceived learning outcomes, and student learning performance within the context of generative artificial intelligence tools such as ChatGPT. A quantitative approach was chosen because of its ability to test theoretically grounded relationships among latent constructs and to produce generalizable insights across a relatively large population.

The target population consisted of undergraduate medical students enrolled in higher education institutions in Punjab, Pakistan. Participants were recruited through a convenience sampling method, commonly used in educational technology research where access to respondents is limited by institutional and logistical factors. Eligibility criteria required participants to (a) be currently enrolled in a medical or allied health program, (b) be willing to participate voluntarily, and (c) have prior experience using GAT, such as ChatGPT, for academic or learning-related purposes. This criterion ensured that respondents had sufficient experience to provide informed evaluations of AI-enhanced learning environments.

### 3.2 Participants

A total of 326 undergraduate students enrolled in medical and allied health education programs constituted the final sample. Of these, 51.5% (*n* = 168) were male and 48.5% (*n* = 158) were female, reflecting a fairly balanced gender distribution. Most respondents were aged 21–23 years (42.6%), followed by those aged 24–26 years (24.8%), indicating that the sample mainly consisted of young adults (Table 1).

**Table 1:** Participants' information.

Variable	Category	Frequency (*n*)	Percentage %
Gender	Male	168	51.5
	Female	158	48.5
Age (years)	18–20	74	22.7
	21–23	139	42.6
	24–26	81	24.8
	27 and above	32	9.8
Program of study	MBBS	173	53.1
	BDS	61	18.7
	Nursing	49	15.0
	Allied health sciences	43	13.2
Year of study	1st year	67	20.6
	2nd year	83	25.5
	3rd year	79	24.2
	4th year	62	19.0
	Final year	35	10.7
Institution type	Public	197	60.4
	Private	129	39.6
Enrollment status	Full-time	308	94.5
	Part-time	18	5.5
Mode of instruction	On-campus	214	65.6
	Hybrid	78	23.9
	Online	34	10.4
Clinical exposure	Pre-clinical	146	44.8
	Clinical	180	55.2

Regarding academic programs, most participants were enrolled in MBBS programs (53.1%), followed by BDS (18.7%), Nursing (15.0%), and Allied Health Sciences (13.2%). Distribution across years of study was fairly balanced, with the highest percentage in the second year (25.5%). More than half of the respondents were in the clinical training stage (55.2%), while 44.8% were in the pre-clinical phase. Additionally, the majority of participants attended public sector institutions (60.4%), studied full-time (94.5%), and primarily received on-campus instruction (65.6%), with some experiencing hybrid or fully online modes.

### 3.3 Instrument

Data were collected using a structured self-administered questionnaire designed to ensure content validity and theoretical alignment with the study framework. The instrument incorporated validated measurement scales adapted from prior empirical studies, thereby enhancing construct reliability and validity. All items were measured using a five-point Likert scale, ranging from 1 (“strongly disagree”) to 5 (“strongly agree”), allowing for the assessment of latent constructs through statistical modeling.

Technology self-efficacy was measured using items adapted from the multidimensional framework developed by Compeau and Higgins (56), reflecting students' confidence in their ability to effectively use AI-based technologies. Cognitive engagement was assessed using a six-item scale derived from Wang et al. (57) and Duncan and McKeachie (58), capturing students' mental effort, persistence, and active involvement in learning tasks. Perceived learning outcomes were measured using five items adapted from Shaofeng et al. (17), representing students' perceived improvement in understanding, knowledge acquisition, and learning effectiveness. Fairness and ethics were assessed using five items adapted from Bernabei et al. (59), focusing on perceptions of equity, transparency, and ethical considerations in AI-supported learning environments. Students' evaluations of Generative AI technologies were measured using six items adapted from prior studies (60), capturing students' interaction with and evaluation of AI tools such as ChatGPT.

Student learning performance in this study refers to self-reported academic effectiveness, including perceived understanding, task efficiency, knowledge acquisition, and the ability to apply learned concepts. It does not represent objective academic grades, but rather reflects students' perceived learning outcomes, consistent with prior research in educational technology (17).

All measurement scales were slightly adapted to align with the context of generative AI in medical higher education. To ensure clarity, relevance, and contextual appropriateness, the instrument underwent a pilot testing and expert validation process. An initial version of the questionnaire was reviewed by university graduates with research expertise, and feedback was collected through structured evaluations and follow-up discussions. Based on this feedback, revisions were made to address ambiguous wording, improve item clarity, and enhance contextual relevance (61, 62).

The final instrument demonstrated strong face validity and content validity, and subsequent statistical analysis confirmed its reliability and construct validity, with Cronbach's alpha and composite reliability values exceeding recommended thresholds.

### 3.4 Ethical approval and informed consent

This study was conducted in accordance with the Declaration of Helsinki and relevant ethical guidelines for research involving human participants. Ethical approval was obtained from the appropriate institutional review board before data collection. Participation was voluntary, and informed consent was obtained electronically from all participants prior to completing the survey. Respondents were assured of their anonymity and confidentiality; no personally identifiable information was collected, and data were used solely for academic research purposes. Participants were informed of their right to withdraw at any time without consequences.

### 3.5 Data collection procedure

A convenience sampling strategy was employed due to accessibility constraints across institutions. Data were collected through an online survey administered during the academic term using a platform such as Google Forms. The survey link was distributed to approximately 400 eligible participants through institutional mailing lists and student communication channels. The survey remained open for 6 weeks, during which four reminders were sent to enhance the response rate. Participation was voluntary, and no monetary incentives were provided.

A total of 354 responses were received, yielding a response rate of 88.5% based on the number of invitations distributed. After screening for incomplete and invalid responses, 326 questionnaires were retained for final analysis. The final sample size is adequate for PLS-SEM analysis, exceeding the recommended minimum threshold of 10 times the maximum number of structural paths directed at any construct (63).

Prior to participation, respondents were informed about the purpose of the study, and assurances were provided regarding anonymity and confidentiality. Informed consent was obtained electronically before questionnaire completion. The online data collection approach enabled efficient access to participants across institutions while ensuring standardized data collection procedures.

Although self-reported survey data provide valuable insights into students' perceptions and experiences, they are subject to potential limitations such as social desirability bias and response consistency effects. To mitigate these issues, anonymity was strictly maintained, respondents were informed that there were no right or wrong answers, and all items were presented in a neutral and non-evaluative manner.

### 3.6 Data analysis techniques

Data analysis was conducted using SmartPLS 4.0, following a two-step approach appropriate for variance-based structural equation modeling (PLS-SEM). Prior to hypothesis testing, descriptive statistics were computed to summarize participants' demographic characteristics and response distributions. The dataset was also screened for missing values, outliers, and normality to ensure data quality and suitability for analysis.

In the first stage, the measurement model was evaluated to assess reliability and validity. Internal consistency reliability was examined using Cronbach's alpha and composite reliability. Convergent validity was assessed through the average variance extracted (AVE), with values exceeding the recommended threshold. Discriminant validity was evaluated using both the heterotrait–monotrait ratio (HTMT) and the Fornell–Larcker criterion, ensuring that each construct was empirically distinct.

In the second stage, the structural model was assessed to test the hypothesized relationships among the study variables. Path coefficients (β), *t-values*, and *p-values* were estimated to evaluate the significance of direct, mediating, and moderating effects. A bootstrapping procedure with 5,000 resamples was employed to assess statistical significance and ensure the robustness of estimates.

Moderation effects were examined using interaction terms, following established PLS-SEM procedures. The explanatory power of the model was evaluated using R^2^ values, while effect sizes and the overall predictive relevance of the model were also considered to assess model adequacy.

## 4 Results

The results are presented in alignment with the study hypotheses. First, measurement model results are reported to establish reliability and validity. Next, structural relationships are examined to test the direct, mediating, and moderating hypotheses.

### 4.1 Measurement model

**Figure 2:**
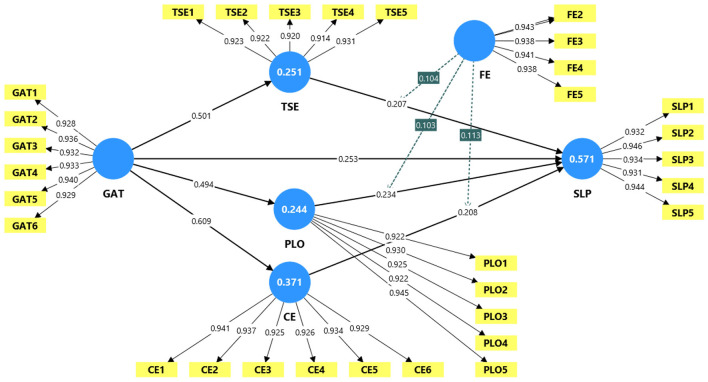
Measurement model.

The measurement model (Table 2, Figure 2) shows high internal consistency, convergent validity, and indicator reliability across all constructs. Cronbach's alpha coefficients range from 0.956 to 0.970, exceeding the recommended threshold of 0.70 and indicating strong internal consistency reliability (64). Additionally, construct reliability is further validated through composite reliability indices (ρA and ρC), both exceeding 0.95, which reflects high measurement stability and robustness (63).

**Table 2:** Measurement model.

Constructs	Items	Value for factor loading	Cronbach's alpha	Composite reliability (rho_a)	Composite reliability (rho_c)	Average variance extracted (AVE)
Cognitive engagement	CE1	0.941	0.970	0.970	0.975	0.868
	CE2	0.937				
	CE3	0.925				
	CE4	0.926				
	CE5	0.934				
	CE6	0.929				
Fairness and ethics	FE1	0.943	0.966	0.970	0.974	0.881
	FE2	0.932				
	FE3	0.938				
	FE4	0.941				
	FE5	0.938				
Generative AI-based technologies	GAT1	0.928	0.970	0.971	0.976	0.871
	GAT2	0.936				
	GAT3	0.932				
	GAT4	0.933				
	GAT5	0.940				
	GAT6	0.929				
Perceived learning outcomes	PLO1	0.922	0.960	0.960	0.969	0.862
	PLO2	0.930				
	PLO3	0.925				
	PLO4	0.922				
	PLO5	0.945				
Student learning performance	SLP1	0.932	0.966	0.966	0.973	0.879
	SLP2	0.946				
	SLP3	0.934				
	SLP4	0.931				
	SLP5	0.944				
Technology self-efficacy	TSE1	0.923	0.956	0.957	0.966	0.850
	TSE2	0.922				
	TSE3	0.920				
	TSE4	0.914				
	TSE5	0.931				
	FE × CE	1.000				
	FE × PLO	1.000				
	FE × TSE	1.000				

CE, cognitive engagement; FE, fairness and ethics; GAT, generative AI technologies; PLO, perceived learning outcomes; SLP, student learning performance; TSE, technology self-efficacy.

Convergent validity is well established, as the average variance extracted (AVE) values for all constructs range from 0.850 to 0.881, significantly exceeding the minimum criterion of 0.50. This demonstrates that a substantial portion of the variance in the observed indicators is explained by their respective latent constructs (64). Additionally, all indicator loadings are positive and exceed 0.914, providing strong evidence of indicator reliability and supporting the adequacy of item-to-construct relationships.

The explanatory power of the model is further supported by the adjusted R^2^ values of the endogenous constructs, which range from 0.241 to 0.560. These values indicate a substantial level of variance explained and meet established benchmarks for model adequacy within the PLS-SEM framework (65). Additionally, the interaction terms representing the moderation effects (FE × CE, FE × PLO, and FE × TSE) exhibit loadings of 1.000, confirming the methodological soundness and statistical appropriateness of the moderation analysis (64).

Overall, the results offer strong evidence of the measurement model's reliability and validity, forming a solid empirical basis for future analysis of the structural relationships.

### 4.2 Discriminant validity HTMT

Discriminant validity was assessed using the heterotrait–monotrait ratio of correlations (HTMT), which is widely recognized as a reliable criterion for assessing construct distinctiveness in variance-based structural equation modeling. As shown in Table 3, none of the HTMT values approach the conservative threshold of 0.85, nor do they surpass the more lenient cutoff of 0.90, providing strong evidence of adequate discriminant validity for all constructs (63, 66).

**Table 3:** Discriminant validity.

Constructs	CE	FE	GAT	PLO	SLP	TSE	FE x PLO	FE x TSE	FE x CE
CE									
FE	0.028								
GAT	0.627	0.031							
PLO	0.287	0.023	0.511						
SLP	0.527	0.223	0.598	0.510					
TSE	0.346	0.042	0.519	0.281	0.478				
FE × PLO	0.035	0.092	0.019	0.119	0.227	0.043			
FE × TSE	0.028	0.020	0.079	0.043	0.164	0.010	0.307		
FE × CE	0.091	0.050	0.021	0.034	0.184	0.035	0.278	0.338	

CE, cognitive engagement; FE, fairness and ethics; GAT, generative AI technologies; PLO, perceived learning outcomes; SLP, student learning performance; TSE, technology self-efficacy.

The HTMT values among the principal latent constructs range from 0.023 to 0.627, indicating that the constructs are empirically distinct and capture conceptually different dimensions of GAI-enhanced learning in medical education. Furthermore, the interaction constructs (FE × PLO, FE × TSE, and FE × CE) show consistently low HTMT values both with the main constructs and among themselves. This pattern is expected from a methodological perspective and appropriate statistically when moderation effects are modeled using a two-stage approach in PLS-SEM, thus confirming the discriminant validity of the interaction terms (64).

Overall, the HTMT results offer strong evidence that all constructs represent distinct theoretical phenomena, thereby confirming the validity of subsequent interpretations of the structural model and the strength of the hypothesized relationships.

### 4.3 Fornell-Larcker criterion

Discriminant validity was further evaluated using the Fornell–Larcker criterion, as shown in Table 4. According to this criterion, the square root of the average variance extracted (AVE) for each construct should be greater than the correlations with other constructs. The results show that, for all constructs, the diagonal values are higher than their correlations with other latent variables, confirming that the Fornell–Larcker criterion is met (64, 67). These findings show that each construct shares a larger portion of variance with its own indicators than with other constructs in the model, providing clear evidence of adequate discriminant validity. Although small negative correlations were observed between FE and several constructs, these do not weaken discriminant validity, as the Fornell–Larcker assessment is based on the magnitude rather than the direction of correlations (63).

**Table 4:** Fornell-Larcker criterion.

Constructs	CE	FE	GAT	PLO	SLP	TSE
CE	0.932					
FE	0.017	0.939				
GAT	0.609	−0.029	0.933			
PLO	0.277	−0.002	0.494	0.929		
SLP	0.510	0.216	0.580	0.492	0.938	
TSE	0.335	−0.035	0.501	0.270	0.460	0.922

CE, cognitive engagement; FE, fairness and ethics; GAT, generative AI technologies; PLO, perceived learning outcomes; SLP, student learning performance; TSE, technology self-efficacy.

Overall, the results confirm that the constructs are empirically and conceptually distinct, thereby supporting the robustness of subsequent structural model estimation and hypothesis testing.

### 4.4 Structural model

**Figure 3:**
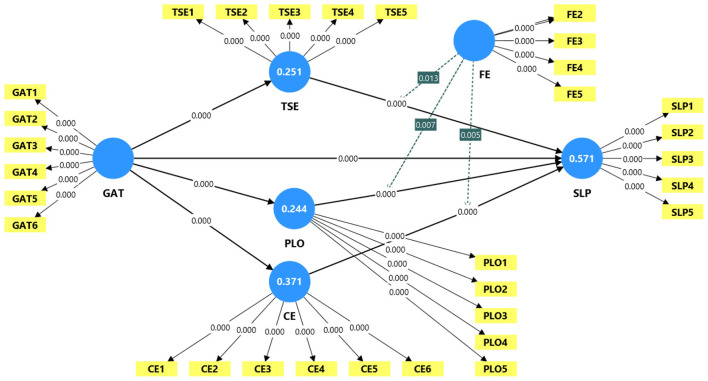
Structural model.

The structural model (Figure 3) was assessed using partial least squares structural equation modeling (PLS-SEM) to test the hypothesized relationships among the study constructs. As shown in Table 5, all structural paths have *t-values* above the critical threshold of 1.96 and *p-values* below 0.05, indicating that the proposed relationships are statistically significant and providing strong empirical support for the hypothesized model (64).

**Table 5:** Path coefficient.

Paths	Original sample (O)	Sample mean (M)	Standard deviation (STDEV)	T statistics (|O/STDEV|)	*p-values*
CE → SLP	0.208	0.208	0.050	4.128	0.000
FE → SLP	0.223	0.223	0.039	5.680	0.000
FE × CE → SLP	0.113	0.112	0.040	2.834	0.005
FE × PLO → SLP	0.103	0.104	0.038	2.696	0.007
FE × TSE → SLP	0.104	0.103	0.042	2.475	0.013
GAT → CE	0.609	0.609	0.037	16.289	0.000
GAT → PLO	0.494	0.494	0.038	13.134	0.000
GAT → SLP	0.253	0.250	0.055	4.564	0.000
GAT → TSE	0.501	0.502	0.039	12.719	0.000
PLO → SLP	0.234	0.236	0.044	5.287	0.000
TSE → SLP	0.207	0.210	0.043	4.844	0.000

CE, cognitive engagement; FE, fairness and ethics; GAT, generative AI technologies; PLO, perceived learning outcomes; SLP, student learning performance; TSE, technology self-efficacy.

### 4.5 Direct effects

The results show that GAT has a strong and positive impact on key learning-related mechanisms, including cognitive engagement (β = 0.609, *p* < 0.001), perceived learning outcomes (β = 0.494, *p* < 0.001), and technology self-efficacy (β = 0.501, *p* < 0.001). Additionally, GAT has a direct positive effect on student learning performance (SLP) (β = 0.253, *p* < 0.001), highlighting the important role of AI-enabled technologies in improving performance outcomes in medical education.

Furthermore, cognitive engagement (β = 0.208, *p* < 0.001), perceived learning outcomes (β = 0.234, *p* < 0.001), and technology self-efficacy (β = 0.207, *p* < 0.001) are all significantly and positively linked to student learning performance, confirming their roles as key mechanisms in the AI-enhanced learning process. Importantly, fairness and ethics show a significant direct impact on student learning performance (β = 0.223, *p* < 0.001), emphasizing the importance of ethical perceptions in shaping educational outcomes in technology-mediated settings.

### 4.6 Moderation effects

Beyond direct relationships, fairness and ethics significantly moderate the effects of cognitive engagement, perceived learning outcomes, and technology self-efficacy on student learning performance. Specifically, the interaction terms fairness and ethics and cognitive engagement FE × CE (β = 0.113, *p* = 0.005), fairness and ethics × perceived learning outcomes (β = 0.103, *p* = 0.007), and FE × TSE (β = 0.104, *p* = 0.013) are all statistically significant. These findings show that the positive effects of AI-enabled learning mechanisms on performance are amplified under higher perceptions of fairness and ethical assurance, which aligns with moderation theory in the PLS-SEM literature (63).

### 4.7 Mediation analysis: specific indirect effects

The mediating mechanisms were further analyzed using bootstrapped indirect effect analysis, with results summarized in Table 6. The findings show that GAT has a significant indirect impact on student learning performance through technology self-efficacy (β = 0.104, *p* < 0.001), perceived learning outcomes (β = 0.116, *p* < 0.001), and cognitive engagement (β = 0.126, *p* < 0.001). All indirect paths have *t-values* greater than 1.96 and *p-values* below 0.05, providing strong evidence of mediation (63, 64).

**Table 6:** Specific indirect path.

Paths	Original sample (O)	Sample mean (M)	Standard deviation (STDEV)	T statistics (|O/STDEV|)	*p-values*
GAT → TSE → SLP	0.104	0.105	0.023	4.514	0.000
GAT → PLO → SLP	0.116	0.116	0.024	4.737	0.000
GAT → CE → SLP	0.126	0.127	0.033	3.854	0.000

CE, cognitive engagement; FE, fairness and ethics; GAT, generative AI technologies; PLO, perceived learning outcomes; SLP, student learning performance; TSE, technology self-efficacy.

These results indicate that the influence of generative AI technologies on learning performance is partly mediated through increased engagement, boosted self-efficacy, and better perceptions of learning outcomes. This supports the theoretical importance of these mediators within the proposed framework.

### 4.8 Total effect

The total effects analysis, which combines both direct and indirect effects, is shown in Table 7. All identified relationships are positive and statistically significant, further confirming the robustness of the proposed model (68). Importantly, the overall effect of GAT on student learning performance is considerable (β = 0.599, *p* < 0.001), demonstrating the combined influence of direct paths and indirect mechanisms through cognitive engagement, perceived learning outcomes, and technology self-efficacy.

**Table 7:** Total effect.

Paths	Original sample (O)	Sample mean (M)	Standard deviation (STDEV)	T statistics (|O/STDEV|)	*p- values*
CE → SLP	0.208	0.208	0.050	4.128	0.000
FE → SLP	0.223	0.223	0.039	5.680	0.000
FE × CE → SLP	0.113	0.112	0.040	2.834	0.005
FE × PLO → SLP	0.103	0.104	0.038	2.696	0.007
FE × TSE → SLP	0.104	0.103	0.042	2.475	0.013
GAT → CE	0.609	0.609	0.037	16.289	0.000
GAT → PLO	0.494	0.494	0.038	13.134	0.000
GAT → SLP	0.599	0.598	0.032	18.834	0.000
GAT → TSE	0.501	0.502	0.039	12.719	0.000
PLO → SLP	0.234	0.236	0.044	5.287	0.000
TSE → SLP	0.207	0.210	0.043	4.844	0.000

CE, cognitive engagement; FE, fairness and ethics; GAT, generative AI technologies; PLO, perceived learning outcomes; SLP, student learning performance; TSE, technology self-efficacy.

Additionally, cognitive engagement (β = 0.208), perceived learning outcomes (β = 0.234), and technology self-efficacy (β = 0.207) all show significant total effects on student learning performance, highlighting their roles as key performance-enhancing factors. Fairness and ethics also have a strong total effect on learning performance (β = 0.223) and significantly strengthen the effects of main predictors through moderation (β range = 0.103–0.113).

### 4.9 Explanatory power (R^2^)

The evaluation of the model's explanatory power was conducted using R^2^ and adjusted R^2^ values, as shown in Table 8. The results demonstrate that GAT account for a moderate proportion of variance in cognitive engagement (*R*^2^ = 0.371) and technology self-efficacy (*R*^2^ = 0.251), as well as a weak-to-moderate proportion in perceived learning outcomes (*R*^2^ = 0.244). Notably, the model explains a substantial proportion of variance in student learning performance (*R*^2^ = 0.571), surpassing commonly accepted benchmarks for strong explanatory power in PLS-SEM (65).

**Table 8:** R-square.

Constructs	R-square	R-square adjusted
CE	0.371	0.369
PLO	0.244	0.241
SLP	0.571	0.560
TSE	0.251	0.248

CE, cognitive engagement; FE, fairness and ethics; GAT, generative AI technologies; PLO, perceived learning outcomes; SLP, student learning performance; TSE, technology self-efficacy.

The close alignment between R^2^ and adjusted R^2^ values shows that the model is stable and not affected by overfitting, which supports its predictive validity (64).

### 4.10 Slope analysis

**Figure 4:**
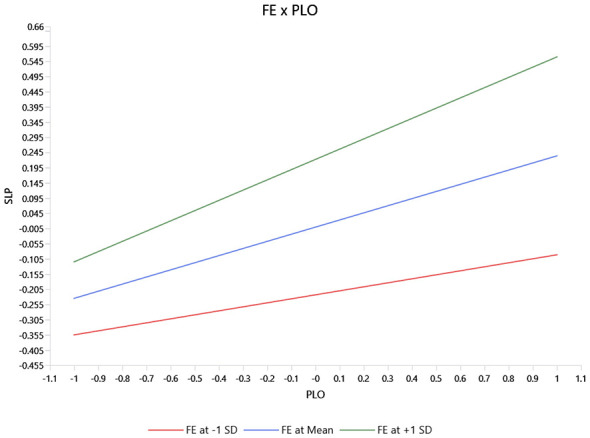
Slope analysis.

To further clarify the moderating role of fairness and ethics, a simple slope analysis was performed, with results shown in Figure 4. The analysis indicates that perceived learning outcomes are positively related to student learning performance at low (−1 SD), mean, and high (+1 SD) levels of fairness and ethics. Notably, the steepest slope appears at high levels of FE, followed by the mean and low levels, suggesting that stronger perceptions of ethics and fairness amplify the positive impact of perceived learning outcomes on performance.

This pattern confirms a positive moderation effect, whereby higher levels of perceived fairness and ethical assurance improve the performance gains connected to AI-supported learning experiences (68, 69).

### 4.11 Model fit

Model fit was evaluated using multiple global fit indices, as shown in Table 9. The standardized root mean square residual (SRMR) scores for both the saturated model (0.026) and the estimated model (0.029) are well below the recommended threshold of 0.08, indicating an excellent fit. The normed fit index (NFI) scores exceed 0.90 for both models, further supporting good comparative fit.

**Table 9:** Model fit.

Features	Saturated model	Estimated model
SRMR	0.026	0.029
d_ULS	0.351	0.455
d_G	0.370	0.363
Chi-square	715.102	686.060
NFI	0.947	0.949

Additionally, the d_ULS and d_G statistics show no signs of model misspecification. Although chi-square values are provided, they are interpreted with caution because chi-square is sensitive to sample size and is not considered a primary fit index in PLS-SEM contexts. Overall, the fit indices confirm that the proposed structural model offers a strong and adequate representation of the observed data (64, 70).

### 4.12 Summary of hypotheses testing

To enhance clarity and facilitate interpretation, the results are summarized in Table 10, which presents each hypothesis, its corresponding relationship, and the overall outcome of hypothesis testing. As shown in Table 10, all proposed hypotheses (H1–H7) were supported, indicating that generative AI technologies influence perceived learning performance both directly and indirectly through key psychological mechanisms, with fairness and ethics strengthening these relationships.

**Table 10:** Summary of hypothesis.

Hypothesis	Statement	Type of effect	Result
H1	Generative AI technologies (GAT) significantly influence student learning performance (SLP).	Direct effect	Supported
H2	Cognitive engagement mediates the relationship between GAT and student learning performance.	Mediation effect	Supported
H3	Perceived learning outcomes mediate the relationship between GAT and student learning performance.	Mediation effect	Supported
H4	Technology self-efficacy mediates the relationship between GAT and student learning performance.	Mediation effect	Supported
H5	Fairness and ethics moderate the relationship between cognitive engagement and student learning performance.	Moderation effect	Supported
H6	Fairness and ethics moderate the relationship between perceived learning outcomes and student learning performance.	Moderation effect	Supported
H7	Fairness and ethics moderate the relationship between technology self-efficacy and student learning performance.	Moderation effect	Supported

## 5 Discussion

This study contributes to the growing body of research on GAI in medical education by providing empirical evidence of how AI-supported learning environments influence students' perceived learning performance through multiple cognitive and psychological mechanisms. The findings indicate that GAI exerts both direct and indirect effects on learning outcomes, mediated by technology self-efficacy, perceived learning outcomes, and cognitive engagement, and moderated by students' perceptions of fairness and ethical considerations.

The results support Social Cognitive Theory, which conceptualizes learning as a dynamic interaction among personal, behavioral, and environmental factors. In this context, GAI functions as an environmental factor that shapes learners' experiences by providing adaptive feedback, personalized instruction, and interactive learning opportunities. These features enhance learners' self-efficacy (personal factor) and cognitive engagement (behavioral factor), which in turn contribute to improved perceived learning effectiveness. The mediating role of self-efficacy aligns with SCT's central premise that individuals who possess stronger beliefs in their capabilities are more likely to engage in complex tasks and achieve favorable outcomes.

The findings further demonstrate that GAI-enhanced educational technologies facilitate personalized and interactive learning experiences, enabling students to engage more deeply with academic content and receive timely, tailored feedback (7, 9). These results are consistent with prior studies highlighting the role of AI in promoting adaptive learning and learner-centered instruction (11, 71). Moreover, the generative and multimodal capabilities of GAI systems contribute to reducing extraneous cognitive load by presenting information in structured and contextually meaningful formats, thereby supporting more efficient cognitive processing (15). In this regard, GAI can be conceptualized not merely as a technological tool, but as a cognitive partner that supports metacognitive regulation and deeper learning processes (72).

The results further emphasize technology self-efficacy as a key psychological mechanism through which generative artificial intelligence impacts academic performance. Consistent with previous studies (16, 17), students who perceive themselves as proficient in using AI technologies tend to exhibit increased confidence, persistence, and a willingness to engage in challenging learning tasks. This supports Bandura's social cognitive theory, which posits that self-efficacy influences beliefs, motivation, and behaviors in learning environments (19, 73). When learners feel capable of effectively navigating AI systems, they are more likely to dedicate sustained effort and actively manage their learning processes. This aligns with Liang et al. (18), who identified self-efficacy and cognitive engagement as sequential mediators between student GAI interaction and academic achievement. Consequently, enhancing students' AI-related self-efficacy should be considered a vital pedagogical strategy to optimize the educational benefits of GAI.

Perceived learning outcomes and cognitive engagement serve as complementary mediators, emphasizing that learning extends beyond mere content delivery to include learners' cognitive involvement and evaluative judgments. GAI enhances learning outcomes by providing immediate, personalized feedback and adaptive learning pathways that support students in monitoring, reflecting on, and regulating their progress (11, 12). These results align with previous research demonstrating improvements in writing proficiency, creativity, and higher-order thinking skills following GAI integration into instructional strategies (74, 75). Furthermore, cognitive engagement is augmented when learners engage in generative, problem-based activities that foster analytical reasoning and creativity, consistent with findings from adaptive learning studies (76). Collectively, these findings illustrate GAI's potential to transition passive learning into active, self-regulated learning, in accordance with social cognitive theory's principle of reciprocal determinism among cognition, behavior, and environment.

A notable contribution of this research is the identification of fairness and ethics as a key moderating factor. Consistent with the findings of Memarian and Doleck (20) and Raza et al. (21), the study demonstrates that students' perceptions of transparency, equity, and ethical practices are crucial in influencing the effectiveness of GAI-enabled learning. Ethical safeguards including unbiased algorithmic design, responsible data management, and transparent content generation, serve to build trust and psychological safety, thereby enhancing the positive impact of engagement, self-efficacy, and perceived learning outcomes on academic performance. Conversely, issues such as algorithmic bias or data misuse can diminish these benefits, highlighting the importance of robust ethical governance in the deployment of AI within educational settings.

From a theoretical perspective, this study extends social cognitive theory by illustrating how triadic reciprocal determinism functions within AI-mediated learning environments. GAI acts as the environmental factor providing instructional scaffolding and external stimuli; technology self-efficacy represents learners' personal cognitive beliefs; and cognitive engagement reflects the behavioral expression of learning through effort and persistence. This integrative framework offers a coherent theoretical and empirically supported explanation of GAI's influence on learning performance, effectively bridging traditional learning theories with contemporary AI-driven pedagogy and contributing to the development of the human and AI co-learning paradigm.

## 6 Policy recommendations

The findings offer strong evidence of GAI's positive impact on student learning outcomes, while also highlighting the importance of responsible and ethically aligned implementation. Policymakers and educational stakeholders should prioritize establishing regulatory frameworks and institutional policies that ensure GAI integration supports broader educational and societal objectives. Transparency, data security, and algorithmic fairness must be regarded as essential principles in deploying GAT (13).

Educational institutions and technology developers should collaborate to establish ethical governance mechanisms that address issues such as data sensitivity, bias mitigation, and accountability (77). Importantly, GAI should be positioned as a complementary instructional resource rather than a substitute for human educators, thereby preserving the central role of teachers in mentoring, contextualizing knowledge, and fostering critical thinking (78). Furthermore, as AI continues to influence educational landscapes worldwide, institutions should incorporate AI literacy and ethical awareness into curricula to equip students and educators with the skills needed to engage critically and responsibly with GAT (79).

## 7 Conclusion

This study provides solid empirical evidence regarding the transformative role of GAT in higher medical education. Based on social cognitive theory, the results illustrate that the effect of GAI on student learning outcomes is mediated by technology self-efficacy, perceived learning achievements, and cognitive engagement, while being significantly influenced by fairness and ethical considerations. The study advances theoretical understanding by shifting the emphasis from adoption focused models to a mechanism based explanation of AI-supported learning outcomes, thereby contributing to empirical research in the relatively underexplored domain of medical education.

From an academic perspective, the findings emphasize that the successful integration of GAI necessitates more than mere technological investment. Higher education institutions must also promote learners' self-efficacy, engagement, and ethical awareness to fully harness the pedagogical advantages of GAI. In conclusion, although GAI offers significant potential to enhance medical education, its efficacy ultimately relies on psychologically prepared learners and the ethical grounding of system design and implementation.

## 8 Limitations and future research

Despite its contributions, this study has several limitations that should be acknowledged. First, the use of a cross-sectional research design limits the ability to establish causal relationships among the studied variables. Future research should employ longitudinal or experimental designs to better examine causal mechanisms and changes in learning over time. Second, the study relies on self-reported survey data, which may be subject to biases such as social desirability and response consistency effects. Although procedural remedies were applied, future studies could incorporate objective measures of learning outcomes, such as academic grades or performance-based assessments, to enhance validity.

Third, the use of a convenience sampling approach and data collection within a specific regional context may limit the generalizability of the findings. Future research should adopt cross-cultural and multi-institutional designs to examine how contextual factors influence the effectiveness of generative AI in diverse educational settings. Fourth, the study focuses on a single population of university students within medical and allied health education, which may restrict broader applicability. Subsequent research should include different disciplines, educational levels, and institutional types to improve external validity.

In addition, this study conceptualizes learning performance as perceived learning effectiveness rather than objective academic achievement. While this approach is consistent with prior educational technology research, future studies should integrate objective performance indicators to provide a more comprehensive understanding of learning outcomes.

Future research may also expand the model by incorporating additional variables such as emotional regulation, learning motivation, instructional design quality, and social support, which may further explain variations in AI-supported learning. Moreover, while this study focuses on generative AI technologies, future investigations should explore other AI systems and emerging technologies to compare their pedagogical effectiveness.

Finally, further research is needed to examine the potential risks and challenges associated with GAI adoption, including overreliance on AI tools, concerns related to academic integrity, and faculty readiness. Addressing these issues will be critical for developing ethical, sustainable, and effective AI integration strategies in medical education.

## Data Availability

The original contributions presented in the study are included in the article/supplementary material, further inquiries can be directed to the corresponding authors.
